# Upper extremity deep vein thrombosis following BBIBP‐CorV COVID‐19 vaccine (Sinopharm vaccine): A case report

**DOI:** 10.1002/ccr3.7535

**Published:** 2023-06-13

**Authors:** Ali Riazi, Masood Faghih Dinevari, Samaneh Abbasian, Amirreza Jabbaripour Sarmadian

**Affiliations:** ^1^ Liver and Gastrointestinal Diseases Research Center Tabriz University of Medical Sciences Tabriz Iran

**Keywords:** BIBP COVID‐19 vaccine, COVID‐19, COVID‐19 vaccines, deep vein thrombosis

## Abstract

**Key Clinical Message:**

In patients with acute symptoms such as pain, swelling, and erythema of the upper extremities shortly after receiving COVID‐19 vaccines, even inactivated virus vaccines, these symptoms may indicate thrombosis, which may be due to the vaccination.

**Abstract:**

BBIBP‐CorV COVID‐19 vaccine (Sinopharm vaccine) is an inactivated whole virus vaccine to control the COVID‐19 pandemic. Studies concluded that inactivated COVID‐19 vaccines do not increase the risk of thrombosis. This report presents a 23‐year‐old man with the chief complaint of severe pain, swelling, and erythema of the right upper extremity following his second dose of the Sinopharm vaccine. Duplex ultrasound of the right upper extremity revealed upper extremity deep vein thrombosis, and treatment started with oral anticoagulation. It is probably the first upper extremity deep vein thrombosis case following inactivated COVID‐19 vaccines.

## 1 INTRODUCTION

Upper extremity deep vein thrombosis (UEDVT) is a rare condition defined as the result of thrombosis in the draining veins of the upper extremities, including the brachiocephalic, subclavian, axillary, brachial, ulnar, and radial veins.^1^ It is characterized by acute onset of upper extremity pain, swelling, erythema, discomfort, and dysfunction.^1^, ^2^ Considering the non‐specificity of the mentioned symptoms, in case of clinical suspicion, it should be confirmed by diagnostic methods such as duplex ultrasound, venography, computed tomography (CT) with contrast, and magnetic resonance imaging (MRI).^3^ Although UEDVT seems benign, it has considerable morbidity and mortality rate, leading to severe conditions such as pulmonary embolism, loss of vascular access, superior vena cava syndrome, and post‐thrombotic syndrome (PTS). Therefore, early diagnosis and prompt initiation of treatment, mainly anticoagulant therapy, should be considered more seriously.^1^, ^2^, ^3^

Several cases of UEDVT have been reported in recipients of the COVID‐19 vaccines.^4^, ^5^, ^6^, ^7^ However, to our knowledge, it has not been reported in recipients of whole inactivated virus COVID‐19 vaccines such as the BBIBP‐CorV vaccine (Sinopharm vaccine). This report presents a 23‐year‐old man with the chief complaint of severe pain, swelling, and erythema of the right upper extremity following his second dose of the Sinopharm vaccine.

## 2 CASE PRESENTATION

A 23‐year‐old right‐hand‐dominant man came to the emergency department with the chief complaint of severe pain, swelling, and erythema of the right upper extremity. He stated that the pain started within 24 hours following his second dose of the Sinopharm COVID‐19 vaccine to his right deltoid muscle about a month before referring to the hospital. At first, the pain was limited to the injection site; after 3 days, he noticed progressive swelling, erythema, and discomfort in his right arm; and a week before referring (21 days after vaccination), the pain and swelling started to intensify in a way that he could not tolerate. So, he self‐referred to the emergency department.

He did not have any past medical history or any history of COVID‐19 disease. He did not use any medication, did not mention any specific familial medical history, and did not have a recent history of surgery or trauma. He was a 2‐pack‐years smoker.

He had a normal state of consciousness and was oriented; His blood pressure was 115/70 mm Hg; his heart rate was 93 bpm; his body temperature was 36.6°C; his respiratory rate was 19; and his oxygen saturation was 97% without supplementation. Physical examinations were performed in the emergency department; the most critical finding was the difference in the size of two limbs by 4 centimeters in the mid‐arm (The right upper limb was more prominent than the left one). He had generalized swelling, erythema, pitting edema, and tenderness in his right arm. The pulse of the right radial artery was relatively weak (+1). He did not have any lymphadenopathy on general examination. Although he did not have COVID‐19 symptoms such as cough, fever, shortness of breath, or fatigue, he received a PCR test due to the current pandemic, which was reported as negative.

The only significant finding of the initial lab test was a slight increase in the D‐dimer level **(**Table 1 **)**, which led us to the clinical suspicion of thrombosis. Therefore, duplex ultrasound of the right upper extremity was requested. The radiologist stated that the axillary vein's distal half and the subclavian vein's entire length are dilated and incompressible, and echogenic thrombosis is seen within them. The diagnosis of right upper extremity deep vein thrombosis is more probable. CT angiography of thoracic arteries was negative for pulmonary embolism, and there was no evidence of mass, lymphadenopathy, or malignancy.

**TABLE 1:** Initial lab tests.

Test	Result	Unit	Reference value
WBC	8.6	×1000/mm^3^	4.0–11
RBC	5.45	×10^6^/mm^3^	M: 4.5–5.8 F: 4–5.2
Hb	16.6	gm/dL	M: 14–18 F: 12–16
Hct	49.3	%	M: 39–52 F: 36–46
MCV	90	FL	80–98
MCH	30	Pgm	27–32
MCHC	34	%	32–36
Platelet	195	×1000/mm^3^	150–450
RDW	13.3	%	11.5–15
PDW	11.7	FL	10–17
MPV	9.5	FL	8.5–12.5
P‐LCR	22.3	%	17–45
INR	1	Index	0.9–1.0
PT	12.5	Sec.	11–13.9
PT ACTIVITY	100	%	70–100
PTT	25	Sec.	25–45
D‐Dimer	440	ng/mL	<250

Although his Wells score was 3, in order to take care of the critical complications, he was admitted to the general internal medicine ward, and treatment was started with oral anticoagulation. Rivaroxaban was prescribed for 6 days in the hospital. Due to the response to treatment, such as improvement in swelling, erythema, and edema, he was discharged on rivaroxaban 15 mg twice daily for 21 days and then 20 mg daily for 3 months.

During hospitalization, several tests were requested to investigate the etiology. Thrombophilia workup was negative, including factor V Leiden, protein C, protein S, anticardiolipin antibody (aCL), anti‐β2 glycoprotein I (aβ2GP1), lupus anticoagulant, and antiplatelet factor 4 (anti‐PF4). However, we did not evaluate prothrombin gene mutation. Since no clear etiology was found for this UEDVT, and the initial symptoms developed shortly after vaccination, this event was probably due to vaccination.

He was followed up for the incidence of PTS at 2 weeks, 3 months, 6 months, and 12 months after discharge using the Villalta scale. In the last follow‐up, the Villalta scale score was evaluated as 4, so he did not suffer from PTS; the symptoms included mild to moderate pain and cramping of the right upper extremity. He was not readmitted after discharge and did not have any critical complaints. As he was right‐handed, his life and job were affected, and his quality of life was disturbed.

## 3 DISCUSSION

COVID‐19 is a multisystem infectious disease affecting several organs and systems, which may lead to thrombotic complications such as venous thromboembolism.^8^ Tan et al.^9^ performed a meta‐analysis study in 2021 on 102 studies involving 64,503 patients and concluded that the frequency rate of COVID‐19‐related venous thromboembolism events was 14.7%–17.6%, and the frequency of COVID‐19‐related arterial thrombotic events was about 3.9%. These events were more common among critically ill patients in intensive care units.^10^

Several vaccines are used around the world to control the COVID‐19 pandemic.^11^ Previous studies concluded that some COVID‐19 vaccines might increase the risks of thrombosis and coagulopathy. In late February 2021, some thrombotic events were reported for the first time in relation to adenoviral vector‐based vaccines, such as ChAdOx1‐S/nCoV‐19 (AstraZeneca vaccine) and Ad26.COV2.S (Johnson & Johnson/Janssen vaccine).^10^ Symptoms often appear 5 days or more following vaccination, commonly severe and persistent headache, abdominal pain, back pain, chest pain with shortness of breath, focal neurological symptoms, and swelling and erythema of extremities.^12^ These events were termed vaccine‐induced immune thrombotic thrombocytopenia (VITT) with five criteria, including the history of recent vaccination, thrombotic complications mostly at unusual sites, thrombocytopenia, increased D‐dimer levels, and positivity of anti‐PF4 antibody. It was more commonly reported in females and patients under 50 years of age; and occurred in various unusual sites, leading to cerebral vein thrombosis (CVT), deep vein thrombosis (DVT), pulmonary thromboembolism (PTE), and splanchnic vein thrombosis (SVT), with significant mortality rate.^10^, ^13^

The BBIBP‐CorV (Sinopharm vaccine), is one of two whole inactivated virus COVID‐19 vaccines that was the fifth authorized vaccine by the World Health Organization (WHO) for emergency use in May 2021 with a 79% efficacy rate. It is authorized by several countries and used widely by the Asian population. Side effects are mostly reported as mild and transient, including dizziness, fatigue, headache, nausea, vomiting, fever, and injection site reactions.^14^, ^15^

Liu et al.^16^ designed and performed a cohort study in 2021 to investigate whether whole inactivated virus COVID‐19 vaccines induce thrombosis. They included 406 healthcare workers who had received two doses, 21 days apart and studied blood samples 4 weeks after the second dose of the vaccine for prothrombotic autoantibodies, including aCL, aβ2GP1, anti‐phosphatidylserine/prothrombin, and anti‐PF4. They concluded that these vaccines do not influence the profile of antiphospholipid and anti‐PF4 antibodies nor increase the risk of thrombosis.

However, Hameed et al.^17^ reported four cases of CVT after receiving inactivated COVID‐19 vaccines (Sinopharm and Sinovac vaccines). Devi et al.^18^ reported a case of VITT presented with pulmonary embolism and thrombocytopenia 2 weeks after receiving the Sinopharm vaccine. Hosseinzadeh et al.^19^ reported another case of VITT presented with evidence of thrombosis in the splenic vein and thrombocytopenia 5 days after receiving the Sinopharm vaccine.

To our knowledge, this is the first report of acute UEDVT following whole inactivated virus COVID‐19 vaccines; other reports occurred after getting mRNA COVID‐19 vaccines, including mRNA‐1273 (Moderna vaccine) and BNT162b2 (Pfizer‐BioNTech vaccine),^4^, ^5^, ^6^, ^7^ which are summarized in Table 2.

**TABLE 2:** Summary of UEDVT cases following COVID‐19 vaccines.

Reference	Patient	Vaccine	Symptoms	Related lab findings	Imaging findings
Bhan et al.^4^	27‐year‐old Female	Second dose of Moderna Vaccine (mRNA‐1273 SARS‐CoV‐2) to the right deltoid muscle	Swelling, pain, and erythema in the right upper extremity	Slight elevation in D‐dimer Levels Normal platelet counts Negative thrombophilia workup	Thrombotic occlusion of the right subclavian and axillary veins by venous duplex ultrasound
Hasegawa et al.^5^	72‐year‐old Female	Second dose of Pfizer‐BioNTech to the left deltoid muscle	Swelling, pain, and dysfunction in the left upper extremity	Normal D‐dimer Levels Normal platelet counts Negative thrombophilia workup	Thrombotic occlusion of the left subclavian and jugular vein by CT
Kang et al.^6^	44‐year‐old Male	Third dose of Moderna Vaccine (mRNA‐1273 SARS‐CoV‐2) to the left deltoid muscle	Swelling, discoloration, and getting hard to touch in the right upper extremity	Slight elevation in D‐dimer levels Normal platelet counts Negative thrombophilia workup	Thrombotic occlusion of the right cephalic arch, axillary, and subclavian vein by venous duplex ultrasound
Gonzalez et al.^7^	60‐year‐old Female	Second dose of Pfizer‐BioNTech to the left deltoid muscle	Swelling, pain, erythema, and warmth in the left upper extremity	Normal D‐dimer Levels Normal platelet counts Negative thrombophilia workup	Thrombotic occlusion of the left internal jugular, subclavian, axillary, and basilic veins by venous duplex ultrasound

Although in the report of Bhan et al.^4^ and Kang et al.,^6^ similar to our case, there is a slight increase in D‐dimer levels, none of these four reports and our case should not be listed in the VITT group; because in all cases, similar to ours, there were negative results of thrombophilia workups, and there was no evidence of thrombocytopenia, so they were in contrast to adenoviral vector COVID‐19 vaccines such as ChAdOx1‐S/nCoV‐19 (AstraZeneca vaccine) and Ad26.COV2.S (Johnson & Johnson/Janssen vaccine).

## 4 CONCLUSION

In conclusion, in patients with acute symptoms such as pain, swelling, and erythema of the upper extremity shortly following COVID‐19 vaccines, even inactivated virus vaccines, imaging, laboratory, and thrombophilia studies should be performed, as these symptoms may indicate thrombosis which may be due to the vaccination. To our knowledge, our case can be introduced as the first upper limb DVT case following inactivated whole virus COVID‐19 vaccine.

## AUTHOR CONTRIBUTIONS

**Ali Riazi:** Conceptualization; data curation; methodology; project administration. **Masood Faghih Dinevari:** Conceptualization; supervision; validation. **Samaneh Abbasian:** Data curation; investigation; validation; writing – review and editing. **Amirreza Jabbaripour Sarmadian:** Investigation; writing – original draft; writing – review and editing.

## FUNDING INFORMATION

There was not any financial support or funding for this research.

## CONFLICT OF INTEREST STATEMENT

The author has no conflict of interest to declare.

## ETHICS STATEMENT

This study was performed according to the principles outlined by the World Medical Association's Declaration of Helsinki on experimentation involving human subjects, as revised in 2000, and has been approved by the ethics committee of the Tabriz University of Medical Sciences with the Approval number IR.TBZMED.REC.1401.159 on 2022/05/11.

## CONSENT STATEMENT

The patient was informed regarding publishing this case report, and written informed consent was obtained in accordance with the journal's patient consent policy.

## Data Availability

Data are available from the corresponding author on reasonable request.
